# The Genetic Contribution to Drug Response in Spondyloarthritis: A Systematic Literature Review

**DOI:** 10.3389/fgene.2021.703911

**Published:** 2021-07-20

**Authors:** Augusta Ortolan, Giacomo Cozzi, Mariagrazia Lorenzin, Paola Galozzi, Andrea Doria, Roberta Ramonda

**Affiliations:** Rheumatology Unit, Department of Medicine DIMED, University of Padova, Padua, Italy

**Keywords:** spondyloarthritis, genes, polymorphism, drug, therapy

## Abstract

**Objective:** Spondyloarthritis (SpA) are a group of diseases with a high heritability, whose pathogenesis is strongly determined by an interplay between genetic and environmental factor. Therefore, the aim of our study was to determine whether genetic variants could also influence response to therapy in SpA.

**Methods:** A systematic literature review (SLR) was conducted in PubMed and Web of Science core collection, without publication-year restrictions (Last search 8th April 2021). The search strategy was formulated according to the PEO format (Population, Exposure, Outcome) for observational studies. The population was adult (≥18 years) patients with SpA. The exposure was inheritable genetic variations of any gene involved in the disease pathogenesis/drug metabolism. The outcome was response to the drug, both as dichotomous (response yes/no) and as continuous outcomes. Exclusion criteria were: (1) languages other than English, (2) case series, case reports, editorials, and reviews, (3) studies reporting genetic contribution to drug response only limited to extra-musculoskeletal features of SpA, (4) epigenetic modifications. Quality of the included study was independently assessed by two authors.

**Results:** After deduplication, 393 references were screened by two authors, which led to the final inclusion of 26 articles, pertinent with the research question, that were considered for qualitative synthesis. Among these, 10 cohort, one cross-sectional, and five case-control studies were considered of at least good quality according to Newcastle-Ottawa Scale (NOS). In studies about TNF-blockers therapy: (1) polymorphisms of the TNF receptor superfamily 1A/1B (*TNFRSF1A/1B*) genes were most frequently able to predict response, (2) −238 and −308 polymorphisms of *TNF*α gene were studied with conflicting results, (3) *TNF*α polymorphism rs1799724, rs1799964, −857, −1,013, +489 predicted drug response in non-adjusted analysis, (4) *PDE3A* rs3794271 had a linear relationship with DAS28 reduction after anti-TNFα therapy. *DHFR* polymorphism +35,289 was able to predict response to methotrexate.

**Conclusions:** Our SLR highlighted the existence of a genetic component in determining drug response. However, further studies are warranted to better define quantify it.

## Introduction

Spondyloarthritis (SpA) is a group of systemic inflammatory diseases with common clinical characteristics and a shared genetic background (Costantino et al., 2018). The typical clinical features include (1) musculo-skeletal manifestations, with axial skeleton (spine and sacroiliac joints) involvement, peripheral arthritis, enthesitis, dactylitis, and (2) extra-musculoskeletal manifestations (EMMs) such as inflammatory bowel disease (IBD), psoriasis, and anterior uveitis. Depending on the main clinical and radiological presentation, the following disease subset have been identified and included under the umbrella term of SpA: ankylosing spondylitis (AS), psoriatic arthritis (PsA), arthritis associated with IBD, reactive arthritis, and undifferentiated SpA (Costantino et al., 2018). Spondyloarthritis have a high heritability, with a complex genetic background that has only been partially elucidated, but which is surely dominated by the Human Leukocyte Antigen (HLA-B27) allele: positive individuals have a relative risk of SpA onset of about 40 compared to those who are HLA-B27 negative. HLA-B27 is part of the Major Histocompatibility Complex class I and it accounts for 20% of the SpA heritability (Costantino et al., 2018). Thus, as strong as its association with the disease might be, HLA-B27 is not the only responsible for SpA genetic susceptibility, as genome wide studies have highlighted in 2007 (Wellcome Trust Case Control Consortium et al., 2007). In particular, among the non-MHC loci, endoplasmic reticulum amino peptidase (*ERAP)1* and Interleukin-23 receptor (*IL23R) genes* were found to be strongly associated with SpA (Wellcome Trust Case Control Consortium et al., 2007). This discovery even led to new pathogenetic hypothesis, with important therapeutic implications (Gaffen et al., 2014).

The importance of genetic factors in the disease susceptibility, prompted researchers to investigate the role of genes in response to therapy as well (Song et al., 2015; Costantino et al., 2018). In fact, heterogeneity in drug response, even with the most effective drugs, has been observed in different disease phenotypes or -in general- in different patients (Ferraccioli et al., 2007). As an example, IL-23 inhibitors are effective in peripheral but not axial manifestations of SpA (Deodhar et al., 2019). Moreover, many patients do not experience adequate disease control with first-line therapy, such as non-steroidal anti-inflammatory drugs or conventional synthetic Disease Modifying Rheumatic Drugs (csDMARDs) and there are no clear indicators to predict this (van der Heijde et al., 2017; Gossec et al., 2020). Furthermore, a consistent proportion of patients (up to one-third) does not even respond to the first biotechnological drug (representing second-line therapy), whichever this might be (Merola et al., 2017). Thus, genetic variants of genes involved in both SpA pathogenesis and phenotypic expression, as well as in the drug metabolism, could play a role in determining drug response (Ferraccioli et al., 2007).

Therefore, the aim of the present study was to collect existing evidence supporting the role of genetics in predicting response to therapy in SpA.

## Materials and Methods

### Literature Search

A systematic literature review (SLR) in accordance with the Preferred Reporting Items for Systematic Reviews and Meta-Analyses guidelines (PRISMA) was conducted (Moher et al., 2009). PubMed and Web of Science core collection were searched, without publication year restrictions. Last search was on 8th April 2021.

The research question was formulated according to the PEO format (Population, Exposure, Outcome) for observational studies. The population (P) of interest was considered to be adult (≥18 years) patients with SpA. Studies including patients with other rheumatic diagnoses were considered eligible only if the results for SpA were presented separately. The exposure (E) was represented by genetic predisposition, meaning specific inheritable genetic variations of any gene that could be involved in the disease pathogenesis, or in drug metabolism. The outcome of interest was drug response, both as dichotomous outcome (response yes/no according to various disease activity status criteria or response criteria) and as continuous outcomes. Examples of dichotomous outcomes were: the Assessment of SpondyloArthritis international Society (ASAS)-based indices ASAS 20, ASAS 40, Bath Ankylosing Spondylitits Disease Activity Index (BASDAI) 50, BASDAI ≥ 4 (Anderson et al., 2001; Rudwaleit et al., 2004). Among continuous outcomes the following were considered: tender/swollen joint count, BASDAI, percentage of patients, Disease Activity Score on 28-joints count (DAS28) (van der Heijde et al., 1990; Garrett et al., 1994).

Inclusion criteria regarding population were: (1) adult axSpA patients as defined by: clinical diagnosis, ASAS criteria for axSpA or modified NY criteria for AS (van der Linden et al., 1984; Rudwaleit et al., 2009); (2) PsA patients as defined by rheumatologist diagnosis or ClAssification criteria for Psoriatic ARthritis (CASPAR) criteria (Taylor et al., 2006); (3) SpA associated to IBD, reactive arthritis or undifferentiated arthritis (if included).

Exclusion criteria were: (1) studies in languages other than English, (2) case series, case reports, editorials, and reviews, (3) studies reporting genetic contribution to drug response only limited to EMMs, such as IBD or psoriasis, and not presenting data for patients with SpA separately, (4) epigenetic modifications (e.g., DNA methylation and miRNA).

We checked MeSH terms for SpA, genetics, drug response to identify search terms in an attempt to capture all possible synonyms. In the final search, however, MeSH terms were not used to avoid excluding more recent works. The detailed search strategy is indicated in the Supplementary File.

### Study Selection, Data Extraction, and Risk of Bias Assessment

Two reviewers (AO, GC) assessed titles and abstracts on suitability for inclusion, according to the inclusion/exclusion criteria, followed by a full-text review if necessary. Discrepancies were resolved by consensus. The following information was extracted from the study: author, year, study design, number of included patients, characteristics of the study population (disease classification, gender, age, disease duration), of the exposure (gene where a variation was detected, and type of variation), and outcome measures. The quality of the extracted studies was then evaluated by Newcastle-Ottawa Scale (NOS) for cross-sectional, cohort, and case-control studies (Wells et al., 2021). Newcastle-Ottawa Scale study quality was then graded according to the total score. Cross-sectional studies were graded as: very good = 9–10; good = 7–8; satisfactory = 5–6; unsatisfactory = 0–4 (Modesti et al., 2016). Cohort and case–control studies were graded as: very good = 8–9; good = 7; satisfactory = 5–6; unsatisfactory = 0–4.

A PRISMA flowchart was generated for the final selection of the studies to be included (see Results section for details).

### Data Extraction

Exposure was expressed as presence or absence of a specific genetic variation. Outcome was expressed according to the analysis presented in the study. If analysis were adjusted, odds ratio (95% Confidence Interval-CI), hazard rate (95%CI), or beta (95%CI) were reported for logistic regression, Cox regression or linear regression, respectively. Otherwise, only *p*-value was reported for descriptive statistics. Due to heterogeneity of the included population, exposure, and outcomes a meta-analysis could not be performed.

## Results

### Study Selection

A total of 524 references were retrieved by the databases search. After removing duplicates, titles, and abstracts of the remaining 393 references were screened for eligibility, which led to the elimination of 330 articles. This was mainly due to wrong target population (e.g., rheumatoid arthritis, psoriasis, gout), wrong exposure (e.g., monocytes expression profile, long non-coding mRNA as inflammatory modulators), or wrong outcome (e.g., disease onset or severity instead of response to therapy); two papers were not in English. The full-text of 61 articles was examined, resulting in the exclusion of 35 further articles that did not fulfill inclusion/exclusion criteria: 29 were reviews or book chapters, one did not present data for SpA separately, one did not specify treatment, four were congress abstracts with insufficient information to extract. The remaining 26 articles were considered for qualitative evaluation.

**Figure 1:**
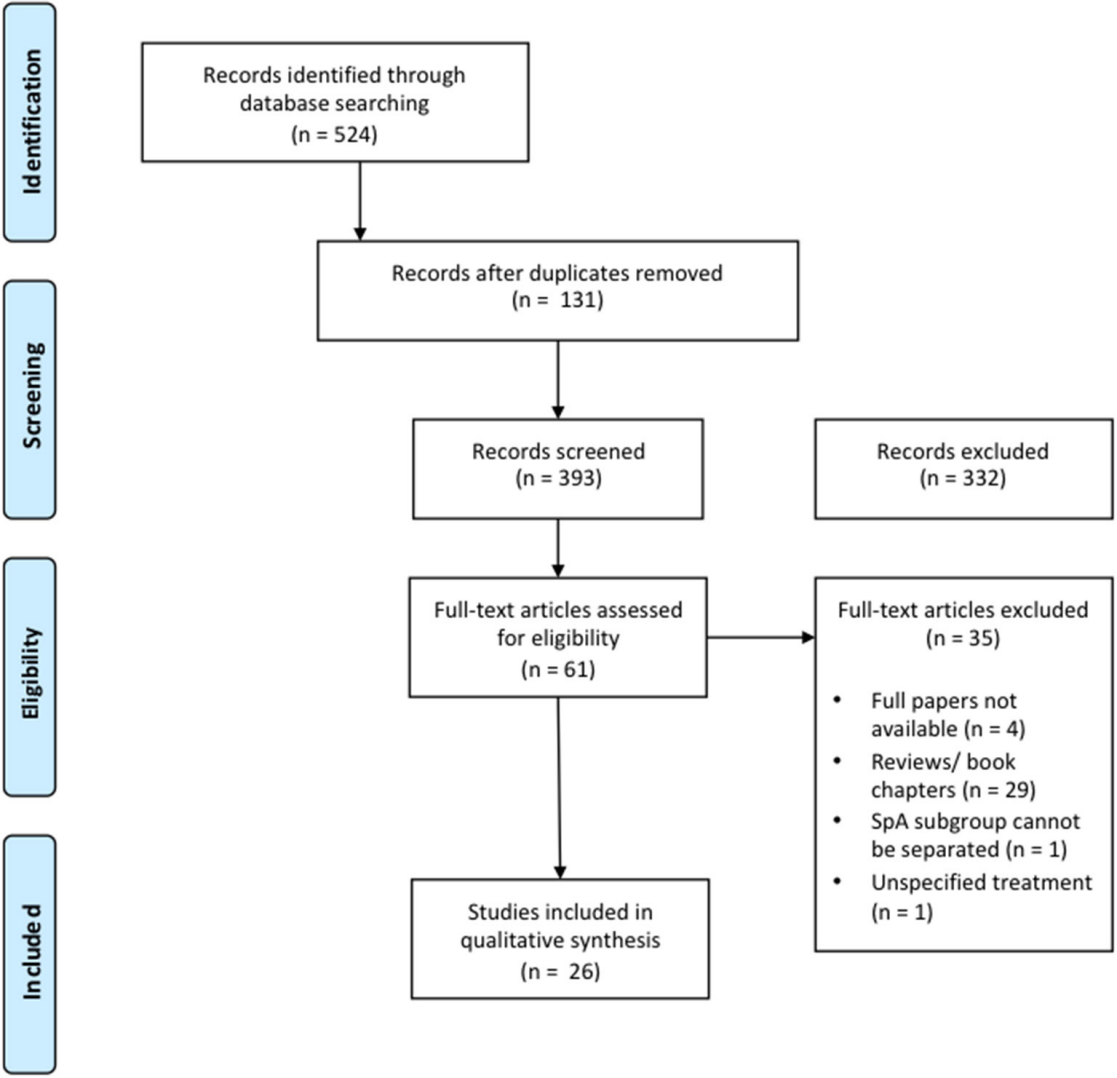
PRISMA flow diagram of studies' inclusion.

The PRISMA flowchart is displayed in Figure 1.

### Study Characteristics

The 26 studies that were included in the qualitative assessment were thoroughly examined to identify: author, year, study design, number of participants, definition of population, exposure, outcome. The main characteristics of the studies are displayed in Table 1. There were 15 cohort studies (Tutuncu et al., 2005; Seitz et al., 2007; Chandran et al., 2010; Eder et al., 2010; Morales-Lara et al., 2010, 2012; Ramírez et al., 2012; Julià et al., 2014; Schiotis et al., 2014; Fabris et al., 2016; Chen, 2017; Yan et al., 2017; Liu et al., 2019; Ovejero-Benito et al., 2019; Polo Y La Borda et al., 2019), eight case-control studies (Manolova et al., 2014; Murdaca et al., 2014; Ma et al., 2017; Wang et al., 2017; Zhao et al., 2017; Aita et al., 2018; Xing-Rong et al., 2018; Xu et al., 2020; Sokolik et al., 2021) and one cross sectional study (Nossent et al., 2014). The definition of the populations was heterogeneous, with studies conducted in Europe, USA, and China, and mainly including AS and PsA patients (Table 1). Exposure was also heterogeneous, as several genetic polymorphisms were evaluated, with target genes implicated in the pathogenesis (e.g., C Reactive Protein—CRP, Tumor Necrosis Factor α–TNFα), drug metabolism (e.g., Cytocrome P450), drug immunogenicity (e.g., Fc receptor). The response to therapy was variably evaluated by validated outcomes of the following types: (1) dichotomous: ASAS 20, ASAS 40, BASDAI 50, American College of Rheumatology (ACR) 20, Psoriatic Arthritis Response Criteria (PsARC) (2) categorical: EULAR response criteria; (3) continuous: tender or swollen joint count, DAS28, BASDAI change score, morning stiffness. Some studies used non-validated but clinically significant outcomes, among which (1) a ≥70% improvement in physician global assessment (PhGA) and SJC/TJC plus a ≥50% improvement in two of: erythrocyte sedimentation rate, CRP, patient global assessment (PGA) (Tutuncu et al., 2005) (2) BASDAI ≤ 4 (Aita et al., 2018) (3) a ≥50% in a Numerical Rating Scale (NRS) for pain (Ovejero-Benito et al., 2019), (4) necessity of therapeutic switch yes/no (Fabris et al., 2016), (5) actively inflamed joint count (meaning tender and/or swollen joints; Chandran et al., 2010).

**Table 1:** Characteristics of the studies satisfying inclusion and exclusion criteria for the SLR, with particular reference to study design and characteristics of population, exposure, outcome.

**References**	**Study** ** design**	**Number of SpA** ** patients**	**SpA** ** subtype**	**Disease definition**	**Males**	**Age ±*SD***	**Country**	**Exposure:** ** candidate** ** gene/s**	**HWE checked**	**Therapy**	**Follow** ** up (weeks)**	**Response to therapy** ** definition**
Xu et al., 2020	Case-control	232	AS	mNY criteria	52,5%	62.3 ± 8.2	China	CRP	Yes, tested variants in HWE	Etanercept	12 w	ASAS20/ASAS40
Morales-Lara et al., 2010	Cohort	49 (33 AS, 16 PsA)	AS/PsA	ND	ND	ND	Spain	Fc receptor	No	Infliximab	48 w	ACR20 or BASFI20
Manolova et al., 2014	Case-control	58	AS	mNY criteria	79,3%	38.1 ± 8.6	Bulgaria	TNFα	Yes, tested variants in HWE	anti-TNFα	24 w	ASAS20
Schiotis et al., 2014	Cohort	121	AS	mNY criteria	73,5%	47.7 ± 9.5	Spain	190 genes among which IL-23 R ERAP 1	Yes, tested variants in HWE	anti-TNFα	12-20 w	BASDAI50
Chen, 2017	Cohort	312	AS	mNY criteria	55,7%	35.2 ± 5.83	China	CYP P450	Yes, tested variants in HWE	Etanercept	24 w	ASAS20, BASDAI50
Morales-Lara et al., 2012	Cohort	55	PsA	CASPAR	56,3%	51.4 ± 10.8	Spain	TNFRSF10A TNFRSF1A	Yes, tested variants in HWE	anti-TNFα	24 w	EULAR criteria
Tutuncu et al., 2005	Cohort	5	PsA	ND	ND	ND	USA	Fc gamma receptor type IIIA	No	anti-TNFα	12 w	≥70% PhGA and SJC/TJC and ≥50% improvement in 2 of: ESR, CRP, PGA, MS
Ramírez et al., 2012	Cohort	103	PsA	CASPAR	52,4%	49.7 ± 13.5	Spain	Fc gamma receptor	No	anti-TNFα	24 w	EULAR criteria
Aita et al., 2018	Case-control	137 (55 AS, 82 PsA)	AS/PsA	mNY criteria / CASPAR	61,3%	51.6 ± 12.6	Italy	TNFα TNF-RSF1A MEFV	Yes, tested variants in HWE	anti-TNFα	144 w	BASDAI ≤ 4
Liu et al., 2019	Cohort	79	AS	mNY criteria and ASAS	88,6%	36.0 ± 11.5	China	MYOM2 VPS13B DISP1 IL27	No	etanercept	12 w	ASAS40
Julià et al., 2014	Cohort	81	PsA	CASPAR	53.0%	48.9 ± 12.7	Spain	PDE3A	No	anti-TNFα	12 w	ΔDAS28
Ovejero-Benito et al., 2019		20	PsA	CASPAR	ND	ND	Spain	TNFRSF1A/1B TNFAIP3 TNIP1 TNF TRAF3IP2	Yes, tested variants in HWE	anti-TNFα	24 w	NRS-Pain50
Polo Y La Borda et al., 2019	Cohort	118 (49 AS, 24 nr-axSpA, 45 p-SpA)	SpA	ASAS	61,8%	53.0 ± 11.2	Spain	36 genes involved mainly in pathogenesis	Yes, tested variants in HWE	anti-TNFα	252 w	Decrease ≥50% or reduction of at least two BASDAI points; EULAR criteria
Xing-Rong et al., 2018Yan et al., 2017	Case-control	215	AS	mNY criteria	82,7%	28.2 ± 9.3	China	TNFRSF1A /1B	Yes, tested variants in HWE	etanerceptSASP celecoxib	48 w	ASAS20, ASAS40
	Cohort	185	AS	mNY criteria	69,1%	37.4 ± 6.2	China	ABCB1	Yes, tested variants in HWE	etanercept	12 w	BASDAI50/ASAS20
Fabris et al., 2016	Cohort	187 (66 AS, 74 nrSpA/pSpA, 47 uSpA)	SpA	ASAS	66,3%	52.0 ± 30.0	Italy	*TNFRSA1B, TNF*α, *FCGR3A, IL-6, IL-6R, TGF*-β	No	anti-TNFα	272 ± 224 w	Non-Switch vs. Switch
Seitz et al., 2007	Cohort	33 (22 AS, 10 PsA)	AS, PsA	mNY criteria, ACR	ND	ND	Switzerland	*TNF*α	No	anti-TNFα	24 w	ΔBASDAI, ΔDAS28
Zhao et al., 2017	Case-control	200	AS	mNY criteria	77,5%	45.8 ± 11.7	China	*TNFRSF1A NLRP3*	Yes, tested variants in HWE	Etanercept, csDMARD	12 w	ASAS20
Tong et al., 2012	Case-control	106	AS	mNY criteria	77,3%	41.6 ± 15.8	China	*TNF*α	Yes, tested variants in HWE	anti-TNFα	12 w	ASAS40-50-70
Murdaca et al., 2014	Case-control	57	PsA	CASPAR	43,8%	50.0 ± 7.0	Italy	*TNF*α	Yes, tested variants in HWE	anti-TNFα	24 w	ASAS20
Nossent et al., 2014	Cross-sectional and cohort	335	AS	mNY criteria	70,1%	45.0± 12.6	Norway	*TNF*α	No	anti-TNFα	340 w (mean)	ΔBASDAI
Ma et al., 2017	Case-control	68	AS		55,8%	32.4 ±12.6	China	*NAT1*	No	MTX	up to 26 w	Morning stiffness, tender joints
Chandran et al., 2010	Cohort	119	PsA	CASPAR	56,3%	44	Canada	*MTHFR* *DHFR* *SLC19A1*	Yes, some variants (rs1051266 and rs180113) were in HWE	MTX	24 w	Actively inflamed joint count
Wang et al., 2017	Case-control	130	AS	mNY criteria	75,3%	30.81 ± 6.92	China	*CYP P450* *COX-2*	No	NSAIDs	12 w	ΔBASDAI, ASAS 20, ASAS40
Eder et al., 2010	Cohort	133	PsA	ND	59,4%	45.6± 12.3	Canada	*MIF*	Yes, tested variants in HWE	IAI	12-24 w	Presence/absence of tenderness or effusion
Sokolik et al., 2021	Case-control	74	PsA	CASPAR	41,8%	46 ± 10.9	Poland	*IL-6*	Yes, tested variants in HWE	MTX	ND	ACR20 PSARC

*SpA, spondyloarthritis; HWE, Hardy-Weingberg equilibrium; AS, ankylosing spondylitis; PsA, psoriatic arthritis; nrSpA, non-radiographic axial spondyloarthritis; pSpA, peripheral spondyloarthritis; uSpA, undifferentiated spondyloarthritis; SD, standard deviation; TNFα, tumor necrosis factor α; IL-23, interleuchin 23; ERAP1, endoplasmic reticulum aminopeptidase 1; CYP P450, cytochrome P450; MYOM2, myomesin 2; VPS13B, vacuolar protein sorting 13 homolog B; DISP1, dispatched RND transporter family member 1; IL-27, interleukin; PDE3A, phosphodiesterase 3A; TNFAIP3, TNFα induced protein 3; TNFRSF1A/1B, Tumor necrosis factor receptor superfamily member 1A/1B; TNFRSF10A, TNF receptor superfamily member 10a; TNIP, TNFα interacting protein 1; TRAF3IP2, TRAF3 interacting protein 2; ABCB1:ATP binding cassette subfamily B member 1; IL-6/IL-6R interleukin 6/ receptor; TGF-β, transforming growth factor β; NLRP3, NLR family pyrin domain containing 3; NAT1, N-acetyltransferase 1; MTHFR, methylenetetrahydrofolate reductase; DHFR, dihydrofolate reductase; SLC19A1, solute carrier family 19 member 1; MIF, macrophage migration inhibition factor; IQR, interquartile range; mNY criteria, modified New York criteria; CASPAR, classification criteria for psoriatic arthritis; ASAS: assessment in ankylosing spondylitis; ACR, American College of Rheumatology; BASFI, bath ankylosing spondylitis function index; BASDAI, bath ankylosing spondylitis disease activity index; EULAR, European league against rheumatism; DAS28, disease activity score for 28 joints; PhGA, physician global assessment; SJC, swollen joint count; TJC, tender joint count; ESR, erythrocyte sedimentation rate; CRP, C-reactive protein; PGA, patient global assessment; MS, morning stiffness; NSR-pain, numeric rating scale for pain; PSARC, psoriatic arthritis response criteria; csDMARDs, conventional synthetic and targeted synthetic; MTX, methotrexate; NSAIDs, non-steroidal anti-inflammatory drugs; IAI, intra articular injection; w, weeks; ND, not defined*.

### Risk of Bias Assessment

According to the NOS for cohort studies, 11 studies were graded as very good or good (Chandran et al., 2010; Eder et al., 2010; Morales-Lara et al., 2012; Ramírez et al., 2012; Julià et al., 2014; Schiotis et al., 2014; Fabris et al., 2016; Chen, 2017; Yan et al., 2017; Liu et al., 2019; Polo Y La Borda et al., 2019), and were therefore included in the qualitative synthesis. One study was deemed unsatisfactory (Morales-Lara et al., 2010) and three were only satisfactory (Tutuncu et al., 2005; Seitz et al., 2007; Ovejero-Benito et al., 2019), thus their results are not discussed in detailed. The lone cross-sectional study was considered of good quality according to NOS (Nossent et al., 2014). Among the case-control studies, four were only satisfactory (Manolova et al., 2014; Wang et al., 2017; Xu et al., 2020; Sokolik et al., 2021), one was unsatisfactory (Ma et al., 2017), and five good (Tong et al., 2012; Zhao et al., 2017; Aita et al., 2018) or very good (Murdaca et al., 2014; Xing-Rong et al., 2018). The latter were the ones that were taken into consideration for the qualitative synthesis. A common reason for higher grades in the cohort studies was the fact that the exposure (genetic polymorphism) was surely present at the start of the study and likely unbiased, resulting from the same laboratory test applied for the whole sample. In general, across all study designs, comparability grading was not always optimal as a minority of studies applied proper correction for several covariates, while the majority only corrected for one important factor or reported unadjusted analysis. Table 2 reports the detailed grading of each study.

**Table 2:** Application of Newcastle-Ottawa quality assessment scale (NOS) for cohort, cross-sectional and case control studies.

**References**	**Score in each Newcastle-Ottawa quality assessment scale item**			**Total score**	**Study quality**
	**Selection**	**Comparability**	**Outcome**		
**Cohort Studies**					
Morales-Lara et al., 2010	1	0	2	3	Unsatisfactory
Schiotis et al., 2014	4	1	3	8	Very good
Chen, 2017	4	1	3	8	Very good
Morales-Lara et al., 2012Tutuncu et al., 2005	4	2	3	9	Very good
	2	1	3	6	Satisfactory
Ramírez et al., 2012	4	1	3	8	Very good
Liu et al., 2019	4	1	2	7	Good
Julià et al., 2014	4	1	3	8	Very good
Ovejero-Benito et al., 2019	2	1	3	6	Satisfactory
Polo Y La Borda et al., 2019Yan et al., 2017	4	1	3	8	Very good
	4	1	3	8	Very good
Fabris et al., 2016	4	3	3	8	Very good
Seitz et al., 2007	3	0	3	6	Satisfactory
Chandran et al., 2010	4	1	3	8	Very good
Eder et al., 2010	4	1	3	8	Very good
	**Selection**	**Comparability**	**Outcome**		
**Cross-sectional studies**					
Nossent et al., 2014	3	1	3	7	Good
	**Selection**	**Comparability**	**Exposure**		
**Case-control studies**					
Xu et al., 2020	2	1	3	6	Satisfactory
Manolova et al., 2014	2	1	3	6	Satisfactory
Aita et al., 2018	3	1	3	7	Good
Xing-Rong et al., 2018	4	2	2	9	Very good
Zhao et al., 2017	3	1	3	7	Good
Tong et al., 2012	3	1	3	7	Good
Murdaca et al., 2014Ma et al., 2017	4	2	3	9	Very good
	0	0	1	1	Unsatisfactory
Wang et al., 2017Sokolik et al., 2021	2	1	3	6	Satisfactory
	3	0	3	6	Satisfactory

*Note of the use of NOS: a cohort study can be awarded a maximum of four stars for the selection category, a maximum of two stars can be given for comparability and a maximum of three stars for the outcome category. A cross-sectional study can be awarded a maximum of five stars for the selection category, a maximum of two stars can be given for comparability and a maximum of three stars for the Outcome category. A case-control study can be awarded a maximum of four stars for the Selection category, a maximum of two stars can be given for comparability and a maximum of three stars for the Exposure category*.

### Result Synthesis

In order to synthetize results regarding the influence of genetic variants on response to therapy, only data from studies that were deemed of good or very good quality were extracted and are presented in Table 3.

**Table 3:** Studies included in the qualitative synthesis.

**References**	**Therapy**	**Follow up**	**Exposure: candidate gene/s**	**Polymorphism**	**Risk genotype/allele**	**Effect size (95%CI) or *p*-value**	**Outcome**	**Effect size adjusted**	**Correction for multiple testing**
Schiotis et al., 2014	Anti-TNFα	12–20 w	*MIF* *IL18RAP* *TNFRSF1B* *ACE* *UQCC1* *ARFGAP2* *ASPN* *CALM1* *IL10* *CYP2D6* *CALM1* *CALM1*	rs755622 rs917997 rs1061622 rs4343 rs6060369 rs3740691 rs331377 rs3213718 rs1800896 rs764481 rs2300496 rs2300500	GG+CG AA+AG GG+TG – – AA+AG – – AA – – –	OR 3.14 (1.19–8.22) OR 3.35 (1.38–8.15) OR 2.46 (1.00–6.04) ns ns OR 2.90 (1.12–7.51) ns ns OR 3.09 (1.04–9.15) – **–** **–**	Non-response according to BASDAI50	Yes, the candidate polymorphism were all included in a multivariate model and effect sizes of independent predictors of non-response are included	No
Chen, 2017	Etanercept	24 w	*CYP2C9* *CYP2D6* *CYP3A5*	rs1057910 rs1065852 rs776746	– CC 3/3	ns *p* < 0.05 vs. CT *p* < 0.05 vs. 1^*^/3^*^	Percentage of responders according to BASDAI50 and/or ASAS20	No	No
Morales-Lara et al., 2012	Anti-TNFα	12–24 w	*TNFRSF10A* *TNFRSF1A*	rs20575 rs767455	– AA	ns *p* = 0.04	EULAR response	No	No
Ramírez et al., 2012	Anti-TNFα	12-24 w	*FCGR2A* *FCGR3A*	rs1801274 rs396991	– RR	ns *p* = 0.03	EULAR response	No	Yes
Aita et al., 2018	Anti-TNFα	40–144 w	*TNF* *TNFRSF1A*	rs1799964 rs1799724 rs1800750 rs1800629 rs361525 rs1800693	– – – – – G	ns ns ns ns ns *p* = 0.03	BASDAI ≤ 4	No	No
Liu et al., 2019	Etanercept	12 w	*MYOM2* *VPS13B* *DISP1* *DISP1* *IL27*	rs2294066 rs7460625 rs2609383 rs2789975 rs17855750	CC – – – –	*p* < 0.0001 ns ns ns ns	ASAS40	No	No
Julià et al., 2014	Anti-TNFα	12 w	*PDE3A*	rs3794271	AA	Beta = −0.71; *p* < 0.0001	ΔDAS28	Yes, for DAS28 baseline value	No
Polo Y La Borda et al., 2019	Anti-TNFα	252 w	*MAPKAPK2* *TLR10* *IRAK3* +other 38 (ref [26])	rs4240847 rs11096957 rs11541076	A T T	HR 1.63 (1.08,2.44) HR 1.49 (1.10,2.04) HR 1.49 (1.00–2.17)	Non response defined as decrease <50% BASDAI or reduction <1.2 of DAS28	No	No
Xing-Rong et al., 2018	Etanercept + sulfasalazyne + colecoxib	48 w	*TNFRSF1A* *TNFRSF1A* *TNFRSF1B*	rs767455 rs2234649 rs1061622	– – TT/GG	ns ns *p* = 0.041 for ASAS20 *p* = 0.021 for ASAS 40	ASAS20 ASAS40	No	No
Yan et al., 2017	Etanercept	12 w	*ABCB1* *ABCB1* *ABCB1*	rs2032582 rs1128503 rs1045642	GG+GA CT+TT –	*p* < 0.05 *p* < 0.05 –	ASAS20 (no differences in ASAS50 e ASAS70)	No	No
Fabris et al., 2016	Anti-TNFα	272 ± 224 w	*TNF* *TNFR2* *IL6* *IL6R* *FCGR3A* *TGF*-β	rs1800629 rs1061622 rs1800795 rs2228145 rs396991 rs19822073	A – GG – – –	OR 4.40 (1.50–13.10) ns ns ns ns ns	EULAR response criteria or BASDAI50 or rheumatologist opinion whether to continue therapy	Yes, covariates were: age, gender, disease duration, diagnosis	No
Zhao et al., 2017	Etanercept, csDMARD	12 w	*TNFRSF1A* *TNFRSF1A* *TNFRSF1A* *TNFRSF1A NLRP3* *NLRP3* *NLRP3* *NLRP3*	rs4149570 rs767455 rs4149569 rs4149621 rs4612666 rs10925019 rs3806265 rs3806268	– – – – – – – G	ns ns ns ns ns ns ns OR 2.17 (1.03-4.56)	ASAS20	Yes, correction for age and gender	No
Tong et al., 2012	Anti-TNFα	12 w	*TNF*	rs1799724 rs1799964 rs1800629 rs361525	T T – –	*p* = 0.0021 *p* = 0.0004 ns ns	ASAS40 and/or ASAS50 and/or ASAS70	No	No
Murdaca et al., 2014	Anti-TNFα	24 w	*TNF*	rs361525 rs1800629 rs80267959	– – G	– – *p* = 0.021^**^	ACR20	No	Yes
Nossent et al., 2014	Anti-TNFα	340 w (mean)	*TNF*	rs361525 rs1800629	– –	ns ns	ΔBASDAI	No	No
Chandran et al., 2010	MTX	24 w	*MTHFR* *DHFR* *DHFR* *SLC19A1*	rs1801131 rs1650697 rs1232027 rs1051266	– A – –	– OR 2.99 (1.20, 7.55) – –	50% reduction in “actively” inflamed joint (tender and/or swollen)	Yes, adjustment for concomitant medication	No
Eder et al., 2010	IAI	12–24 w	*MIF*	rs755622	GG + GC	ns	No tenderness or effusion in the injected joint	Yes, adjustment for age, sex, duration of PsA. disease activity	No

* *Only significant genotypes or risk alleles, among those tested, are indicated*.

** *Nominal p significance*.

*Ns, not significant; OR, odds ratio; IAI, intra articular injection; MTX, methotrexate; ACR, American College of Rheumatology; ASAS, assessment in ankylosing spondylitis; BASDAI, bath ankylosing spondylitis disease activity index; EULAR, European League Against Rheumatism; DAS28, disease activity score for 28 joints; PsA, psoriatic arthritis; MIF, macrophage migration inhibitory factor; IL18RAP, interleukin 18 receptor accessory protein; TNFRSF1B, TNF receptor superfamily member 1B; ACE, angiotensin I converting enzyme; UQCC1, ubiquinol-cytochrome C reductase complex chaperone 1; ARFGAP2, ADP ribosylation factor GTPase activating protein 2; ASPN, asporin; CALM1, calmodulin 1; IL10, interleukin 10; CYP2C9, cytochrome P450 family 2 subfamily C member 9; CYP2D6, cytochrome P450 family 2 subfamily D member 6;CYP3A5, cytochrome P450 family 3 subfamily A member 5; TNFRSF10A, TNF receptor superfamily member 10a; FCGR2A, Fc fragment Of IgG Receptor IIa; FCGR3A, Fc fragment Of IgG receptor IIIa; TNFRSF1A, tumor necrosis factor receptor superfamily member 1A; MEFV, mediterranean fever; MYOM2, myomesin 2; VPS13B, vacuolar protein sorting 13 homolog B; DISP1, dispatched RND transporter family member 1; IL27, interleukin 27; PDE3A, phosphodiesterase 3A; MAPKAPK2, mitogen activated protein kinase activated protein kinase 2; TLR10, Toll-like receptor 10; IRAK-3, interleukin 1 receptor associated kinase 3; TNFRSA1B, tumor necrosis factor receptor superfamily member 1B; ABCB1:ATP binding cassette subfamily B member 1; TNF, tumor necrosis factor alpha; TNFR2, tumor necrosis factor receptor 2; IL6, interleukin 6; IL6R, interleukin 6 receptor; TGF-β, transforming growth factor beta; DHFR, dihydrofolate reductase; NLRP3, NLR family pyrin domain containing 3; MTHFR, methylenetetrahydrofolate Reductase; SLC19A1, solute carrier family 19 member 1; RFC, reduced folate carrier*.

#### Genes Involved in SpA Pathogenesis

Most of the studies focused on anti-TNFα therapy. Several of them investigated genes involved in SpA pathogenetic mechanisms, in particular TNFα, TNFα receptors, and several interleukins (IL) both with pro inflammatory (e.g., IL-6) and anti-inflammatory (e.g., IL-10) effects.

Polymorphisms of the TNF receptor superfamily 1 A and 1B (*TNFRSF1A/1B*) genes were those that most frequently were able to predict response (*TNFRSF1A* rs767455 genotype AA, *TNFRSF1A* rs1800693 genotype GG, *TNFRSF1B* rs1061622 genotype TT and GG) according to various criteria such as BASDAI 50, EULAR response, or ASAS 20, ASAS 40 (Morales-Lara et al., 2010; Julià et al., 2014; Ovejero-Benito et al., 2019). Notably, Schiotis et al., who also investigated the *TNFRSF1B* polymorphism rs1061622, found that the GG genotype was associated with non-response, thus reaching opposite conclusion compared to the previously mentioned studies despite a fair numerosity and correcting for other polymorphisms (Schiotis et al., 2014). Other authors simply could not demonstrate any association to response according to the ASAS 20 for the polymorphisms they investigated in the *TNFRSF1A* gene (rs2234649, rs4149570, rs4149621, rs4149569; Zhao et al., 2017; Xing-Rong et al., 2018). Notably, among the investigated genetic variations, also *TNFRSF1A* rs767455 was present, and its association with clinical response was therefore not confirmed by all authors (Zhao et al., 2017; Xing-Rong et al., 2018).

The TNFα gene was also frequently studied in relation to therapy response, with two studies failing to demonstrate an association of the −238G>A (rs361525) and −308G>A (rs1800629) polymorphisms and clinical response according to ACR 20 and BASDAI (Murdaca et al., 2014; Nossent et al., 2014). These studies, however, did not correct for any confounding factor. Conversely, Fabris et al., correcting the association of the same −308 A polymorphism to therapy response (according to ASAS0 20) for age, gender, disease duration, and diagnosis, found a significantly positive association (Fabris et al., 2016). Other TNFα gene polymorphism described to be associated to either ASAS 40, ASAS 50, or ACR 20 response were −857C>T (rs1799724), −1031T>C (rs1799964), while +489G>A (rs80267959) was associated to ACR 20 response. All these findings derived, however, from non-adjusted analysis and were not confirmed by all studies (Tong et al., 2012; Murdaca et al., 2014; Aita et al., 2018).

Furthermore, genes encoding for molecules implicated in the signaling transduction cascade (including inflammatory cascade), such as phosphodiesterase *(PDE)3A*, were shown to have a linear relationship with DAS28 reduction after anti-TNFα therapy (Julià et al., 2014). Other polymorphisms implicated in SpA pathogenesis that were found to be independently associated to non-response were: rs755622 in macrophage migration inhibition factor (*MIF*), rs917997 in IL18-receptor accessory protein (*IL18-RAP*), rs3740691 in ADP Ribosylation Factor GTPase Activating Protein 2 (*ARFGAP2*), rs1800896 in *IL-10*, 2rs4240847 in Mitogen-Activated Protein Kinase-Activated Protein Kinase (*MAPKAPK2*), rs11096957 in Toll like receptor-10 (*TLR-10*), rs11541076 in Interleukin 1 Receptor Associated Kinase 3 (*IRAK-3*) (Schiotis et al., 2014; Polo Y La Borda et al., 2019).

Finally, one study, among those of good quality investigating pathogenetic genes, explored the role of *MIF* polymorphism rs755622 in predicting clinical response to intra-articular steroid injections in PsA; the analysis failed to show any association when correcting for age, sex, disease duration, and activity (Eder et al., 2010).

#### Genes Involved in Drug Metabolism or Immunogenicity

Fewer authors took into consideration genes that might be involved in drug metabolism or immunogenicity. Amid these, genes encoding for enzymes that are part the cytochrome (CYP) P450 superfamily have been tested: the allele variants CYP2D6^*^10 and CYP3A^*^3 were more frequently found in BASDAI50 responders than non-responders to etanercept (Chen, 2017). Other works examined genes encoding for the Fc fragment receptor 2A and 3A, under the hypothesis that polymorphisms resulting in a higher/lower affinity to the Fc region of TNFα blockers may modulate both their half-life and cellular effects, and may therefore produce differential therapeutic effects in individuals (Ramírez et al., 2012). Ramirez et al. found that *FCGR3A* was indeed associated to EULAR response, although in a non-adjusted analysis (Ramírez et al., 2012). Fabris et al. were not able to confirm this finding after adjusting for age, gender, disease duration, and diagnosis (AS/PsA) (Fabris et al., 2016).

One study investigated response to methotrexate in terms of reduction of at least 50% of “actively inflamed joints,” meaning tender and/or swollen joints, highlighting that *DHFR* polymorphism +35289A>G (rs1232027) was able to predict response to methotrexate (even when correcting for concomitant medications; Chandran et al., 2010).

A synthesis of the genes that have been found to be associated to drug response is represented in Table 4.

**Table 4:** Synthesis of genes that have been studied in relation to treatment response in spondyloarthritis, and summary of results.

**Candidate gene**	**Polymorphism**	**References**	**Risk genotype/Allele**	**Significant association with clinical response to drugs**
*TNF*	rs1799724	Tong et al., 2012	T	Yes, positively associated to ASAS40 and/or ASAS50 and/or ASAS70
		Aita et al., 2018	–	No
	rs1799964	Tong et al., 2012	T	Yes, positively associated to ASAS40 and/or ASAS50 and/or ASAS70
		Aita et al., 2018	–	No
	rs1800629	Tong et al., 2012	–	No
		Fabris et al., 2016	A	Yes, positively associated to EULAR response criteria or BASDAI50 or rheumatologist opinion whether to continue therapy
		Murdaca et al., 2014		No
		Nossent et al., 2014	–	No
		Aita et al., 2018	–	No
	rs361525	Tong et al., 2012	–	No
		Murdaca et al., 2014	–	No
		Nossent et al., 2014	–	No
		Aita et al., 2018	–	No
	rs80267959	Murdaca et al., 2014	G	Yes, positively associated to ACR20
	rs1800750	Aita et al., 2018	–	No
*TNFRSF1A*	rs4149570	Zhao et al., 2017	–	No
	rs767455	Zhao et al., 2017	–	No
		Xing-Rong et al., 2018	–	No
		Morales-Lara et al., 2012	AA	Yes, positively associated to EULAR response criteria
	rs4149569	Zhao et al., 2017	–	No
	rs4149621	Zhao et al., 2017	–	No
	rs2234649	Xing-Rong et al., 2018	–	No
	rs1800693	Aita et al., 2018	G	Yes, positively associated to BASDAI
*TNFRSF1B*	rs1061622	Xing-Rong et al., 2018	TT/GG	Yes, positively associated to ASAS20/ASAS40
		Polo Y La Borda et al., 2019	–	No
		Schiotis et al., 2014	GG+TG	Yes, negatively associated with BASDAI50
	rs3397	Polo Y La Borda et al., 2019	–	No
	rs976881	Polo Y La Borda et al., 2019	–	No
*PDE3A*	rs3794271	Julià et al., 2014	AA	Yes, positively associated to ΔDAS28
		Polo Y La Borda et al., 2019	–	No
*HFR*	rs1650697	Chandran et al., 2010	A	Yes, positively associated to 50% reduction in “actively” inflamed joint (tender and/or swollen)
	rs1232027	Chandran et al., 2010	–	No

*ACR, American College of Rheumatology; ASAS, assessment in ankylosing spondylitis; BASDAI, bath ankylosing spondylitis disease activity index; EULAR, European League Against Rheumatism; DAS28, disease activity score for 28 joints; TNF, tumor necrosis factor alpha; TNFRSF1B, TNF receptor superfamily member 1B; TNFRSF1A, tumor necrosis factor receptor superfamily member 1A; PDE3A, phosphodiesterase 3A; DHFR, dihydrofolate reductase*.

## Discussion

The results of our SLR highlighted that the genetic component is surely one of the determinants of drug response in SpA. However, the heterogeneity existing in present literature prevented us to quantify the genetic contribution to therapy response, particularly regarding anti-TNFα biological drugs, which were the most studied.

Admittedly, there are several challenges in conducting predictions studies about genetic variants in drug response in SpA. Firstly, given that most studies focused on genes involved in the disease pathogenesis, it must be remembered that several pathways have been implied in this process. Dysregulation of the IL-17/23 axis and the activation of innate immunity, with effectors like gamma-delta T cells, type 3 innate lymphoid cells (ILCs), neutrophils, macrophages, and lately also cytotoxic B lymphocytes have been described in SpA (Tang and Inman, 2021). In addition, interaction with environmental triggers is fundamental for disease onset and perpetuation. As an example, polymorphisms of TLR-2 and−4, key receptors in pathogen recognition expressed by macrophages or dendritic cells, have been associated to SpA onset at an early age (Perica et al., 2015). When certain genetic variants are associated to disease onset or severity, it is logical to suspect they might be involved in drug response as well. However, since pathogenesis is not solely driven by one of these mechanisms, it is unlikely that a single gene, or a narrow spectrum of gene within a particular pathway, might significantly explain the tendency to respond to a certain targeted therapy. Furthermore, several aspects of SpA pathogenesis are still unknown: one above all, it is not clear how HLA-B27 exerts its pathogenetic effect. For this reason, comprehensive genetic approaches, such as genome-wide association studies (GWAS) have been undertaken in order to uncover unknown factors of susceptibility (Jung et al., 2014; Robinson et al., 2016). This kind of studies might also have therapeutic implications, and has the advantage, compared to the classic candidate-gene(s) design, of being hypothesis-free. Both candidate and whole genome strategies have limitations, however, candidate gene approach lacks the objectivity of genome-wide screening in the process of choosing specific candidates from numbers of potential possibilities; the choice of genes depends on the prior knowledge of the illness, which often remains partly unknown (Sabourin et al., 2019). In addition, in order to be clinically useful, a quite strong relation between a certain genetic variant and clinical outcomes has to be highlighted. Not to mention the candidate gene should also have a demonstrated added value, compared to clinical predictors of response (e.g., male sex), to be of interest (Ni et al., 2013; Ramonda et al., 2021). In practice, it is often the case that certain polymorphisms are only weakly associated to drug response. This can clearly be seen from the adjusted OR, along with their wide 95% CI, represented in Table 3 (Schiotis et al., 2014; Fabris et al., 2016; Zhao et al., 2017; Polo Y La Borda et al., 2019).

A second, but not less important, issue is represented by the reproducibility of results. Even studies investigating the same polymorphism, such as TNFα −308, which has been associated to anti-TNF response both in adult and juvenile SpA (Scardapane et al., 2012), often have contrasting results (Murdaca et al., 2014; Nossent et al., 2014; Fabris et al., 2016). In part, this could be due to the small sample size of some of these studies or to the diversities in the included ethnicities (e.g., Asian vs. Caucasian). On the other hand, the outcomes of drug response are also not standardized across studies. Moreover, analysis are carried out very differently, adjusting for different sets of factors, or without any/with very little adjustment. All these factors add up to the challenge of detecting significant and reproducible genetic markers of drug response.

Thirdly, it has been highlighted how genetic research is particularly prone to type I error (i.e., the risk of falsely rejecting a true null hypothesis or, in other words, to identify a significant association when indeed no association exists; Sabourin et al., 2019). This might happen because of the highly non-independent nature of the variants in a genome, which implies that the assumptions underlying the commonly used statistical methods are often not met (Sabourin et al., 2019). Furthermore, more commonly type I error may stem from multiple testing (comparison of several variants), genotyping errors, and population stratification, that can result in spurious associations (Jorgensen et al., 2009). One of the most obvious, yet important, remedies for this, would be to correct for multiple testing, especially in the candidate-gene approach studies where several variants are tested. Unfortunately, only a slight minority of the retrieved studies applied this correction (Table 3), although this might be less impactful in those studies which tested a limited number of variants (e.g., 3–4). Another way it has been found to limit this problems is replication or cross-validation within the same sample (Liu et al., 2019).

Certainly, however, the fact that several polymorphisms, mainly implicated in the disease pathogenesis, were able to predict to some extent the treatment response, even in adjusted analysis and with a fair numerosity in study populations, points toward the real existence of a genetic determination of drug response (Julià et al., 2014; Schiotis et al., 2014; Fabris et al., 2016; Zhao et al., 2017). This was especially seen with TNFα-blockers therapy, which is also the most frequently used effective therapy for SpA (van der Heijde et al., 2017). Studies investigating polymorphisms involved in drug metabolism in anti-TNFα were less consistent. Interestingly, also response to methotrexate seemed to be predicted by a polymorphism of a gene involved in drug metabolism (*DHFR* +35289), which is somehow more expected than for anti-TNFα as methotrexate is a traditional csDMARD, with a prevalent liver metabolism.

Our study had the methodological strength of being a SLR, and therefore we were able to capture all relevant literature pertaining our research questions, as well as providing a quality assessment of each study. The potential limitations are linked to the design of included studies, which all used a candidate-gene approach: this kind of research is more prone to type I error and to publication bias (i.e. the presentation of mostly positive results, neglecting studies with negative findings). To this regard, GWAS studies could be at a lower risk of bias. Moreover, no RCT taking genetic variants into consideration was retrieved, but only observational studies. Other issues were heterogeneity in the description of population, exposure and outcome. The latter prevented us to perform a meta-analysis to quantify the genetic contribution to drug response in SpA.

In conclusion, we were able to identify a genetic component in drug response across all the included study. Incorporating genetic analysis into clinical studies could help to predict responses to different treatment options, aiming toward personalized medicine. However, further studies are warranted to better define the genotypes that are most involved in contributing to response to therapy and to describe the magnitude of this phenomenon, especially in comparison with the most commonly used clinical predictors.

## Data Availability Statement

The original contributions presented in the study are included in the article/Supplementary Material, further inquiries can be directed to the corresponding author/s.

## Author Contributions

AO and GC participated in study design, data extraction, analysis and synthesis, and drafted the manuscript. ML and PG helped in data collection, critical interpretation of data, and revised the manuscript for important intellectual content. AD and RR conceived the study, analyzed the results critically, and revised the manuscript for important intellectual content. All authors approved the final version to be published.

## Conflict of Interest

The authors declare that the research was conducted in the absence of any commercial or financial relationships that could be construed as a potential conflict of interest.
