# Supplementary material for: The Genetic Contribution to Drug Response in Spondyloarthritis: A Systematic Literature Review
Source: Front Genet. 2021 Jul 20;12:703911. doi: 10.3389/fgene.2021.703911 (PMC8329488; doi:10.3389/fgene.2021.703911)
Supplement: Supplementary file 1 [file Data_Sheet_1.docx]

**Literature search strategy**

**PubMed**

(Spondylarthritis) OR (Spinal Arthritis) OR (Spinal Arthritides) OR (Arthritis, Spinal) OR (Psoriasis, Arthritic) OR (Arthritic Psoriasis) OR (Psoriatic Arthritis) OR (Psoriasis Arthropathica) OR (Psoriatic Arthropathy) OR (Arthropathies, Psoriatic) OR (Arthropathy, Psoriatic) OR (Psoriatic Arthropathies) OR (Spondyloarthritis Ankylopoietica) OR (Ankylosing Spondylarthritis) OR (Ankylosing Spondylarthritides) OR (Spondylarthritides, Ankylosing Spondylarthritides, Ankylosing) OR (Spondylarthritis, Ankylosing) OR (Ankylosing Spondylitis) OR (Spondylarthritis Ankylopoietica) OR (Bechterew Disease) OR (Bechterew's Disease) OR (Bechterews Disease) OR (Marie-Struempell Disease) OR (Marie Struempell Disease) OR (Rheumatoid Spondylitis) OR (Spondylitis, Rheumatoid) OR (Spondylitis Ankylopoietica) OR (Ankylosing Spondyloarthritis) OR (Ankylosing Spondyloarthritides) OR (Spondyloarthritides, Ankylosing) OR (Spondyloarthritis, Ankylosing)

(therapeutic response) OR (therapy response) OR (clinical response) OR (BASDAI) OR (Bath Ankylosing Spondylitis Disease Activity Index ) OR (ASDAS) OR (Ankylosing Spondylitis Disease Activity Score) OR (Disease Activity Psoriatic Arthritis ) OR (DAPSA) OR (Composite Psoriatic Disease Activity Index) OR (CPDAI)

(Polymorphisms, Genetic) OR (Genetic Polymorphism) OR (Genetic Polymorphisms) OR (Gene Polymorphism) OR (Gene Polymorphisms) OR (Polymorphism, Gene) OR (Polymorphisms, Gene) OR (Polymorphisms (Genetics)) OR (Polymorphism (Genetics))

#1 AND #2 AND #3 (167 risultati)

**Web of Science**

((TS=Spondylarthritis) OR (TS=Spinal Arthritis) OR (TS=Spinal Arthritides) OR (TS=Arthritis, Spinal) OR (TS=Psoriasis, Arthritic) OR (TS=Arthritic Psoriasis) OR (TS=Psoriatic Arthritis) OR (TS=Psoriasis Arthropathica) OR (TS=Psoriatic Arthropathy) OR (TS=Arthropathies, Psoriatic) OR (TS=Arthropathy, Psoriatic) OR (TS=Psoriatic Arthropathies) OR (TS=Spondyloarthritis Ankylopoietica) OR (TS=Ankylosing Spondylarthritis) OR (TS=Ankylosing Spondylarthritides) OR (TS=Spondylarthritides, Ankylosing Spondylarthritides, Ankylosing) OR (TS=Spondylarthritis, Ankylosing) OR (TS=Ankylosing Spondylitis) OR (TS=Spondylarthritis Ankylopoietica) OR (TS=Bechterew Disease) OR (TS=Bechterew's Disease) OR (TS=Bechterews Disease) OR (TS=Marie-Struempell Disease) OR (TS=Marie Struempell Disease) OR (TS=Rheumatoid Spondylitis) OR (TS=Spondylitis, Rheumatoid) OR (TS=Spondylitis Ankylopoietica) OR (TS=Ankylosing Spondyloarthritis) OR (TS=Ankylosing Spondyloarthritides) OR (TS=Spondyloarthritides, Ankylosing) OR (TS=Spondyloarthritis, Ankylosing)) AND

((TS=therapeutic response) OR (TS=therapy response) OR (TS=clinical response) OR (TS=BASDAI) OR (TS=Bath Ankylosing Spondylitis Disease Activity Index ) OR (TS=ASDAS) OR (TS=Ankylosing Spondylitis Disease Activity Score) OR (TS=Disease Activity Psoriatic Arthritis ) OR (TS=DAPSA) OR (TS=Composite Psoriatic Disease Activity Index) OR (TS=CPDAI)) AND ((TS=Polymorphisms, Genetic) OR (TS=Genetic Polymorphism) OR (TS=Genetic Polymorphisms) OR (TS=Gene Polymorphism) OR (TS=Gene Polymorphisms) OR (TS=Polymorphism, Gene) OR (TS=Polymorphisms, Gene) OR (TS=Polymorphisms (Genetics)) OR (TS=Polymorphism (Genetics)))
